# Response of Oxidative Stress and Antioxidant System in Pea Plants Exposed to Drought and Boron Nanoparticles

**DOI:** 10.3390/antiox12020528

**Published:** 2023-02-20

**Authors:** Rūta Sutulienė, Aušra Brazaitytė, Stanisław Małek, Michał Jasik, Giedrė Samuolienė

**Affiliations:** 1 Institute of Horticulture, Lithuanian Research Centre for Agriculture and Forestry, Kaunas Str. 30, Kaunas District, LT-54333 Babtai, Lithuania; 2 Department of Ecology and Silviculture, Faculty of Forestry, University of Agriculture in Krakow, Al. 29-go Listopada 46, 31-425 Kraków, Poland

**Keywords:** pea, drought, boron, nanoparticles, antioxidants, oxidative stress

## Abstract

Pea plants are sensitive to water shortages, making them less attractive to farmers. Hoping to reduce the adverse effects of drought on peas and considering the benefits of boron, this study aimed to investigate the impact of boron nanoparticles on the antioxidant system and oxidative stress biomarkers in drought-stressed peas. Experiments were performed in a greenhouse. Pea plants were treated with a suspension of B_2_O_3_ nanoparticles at 12.5, 25, and 50 ppm concentrations before ten days of water shortage. Drought effects were induced by maintaining 30% substrate moisture. This study investigated the properties of the nanoparticle suspension and different application methods for spraying and watering pea plants. The effects of B_2_O_3_ nanoparticles and drought were determined on pea growth indicators, oxidative stress biomarkers, and enzymatic and non-enzymatic antioxidants. Spraying with B_2_O_3_ nanoparticles at 12.5 ppm most effectively stimulated phenol accumulation; FRAP, DPPH, and ABTS antioxidant capacity; and APX, SOD, GPX, and CAT enzyme activity in pea leaves exposed to drought. In addition, B_2_O_3_ nanoparticles reduced the amount of MDA and H_2_O_2_ in pea plants grown on a substrate with insufficient moisture. The most substantial positive effect was found on peas affected by drought after spraying them with 12.5 ppm of B_2_O_3_ nanoparticles. B_2_O_3_ nanoparticles positively affected the pea height, leaf area, number of nodules, and yield.

## 1. Introduction

Nanotechnology is an advanced field of science, the use of which ranges from standard household chemicals and cosmetics to precision agriculture, the development of medicines and medical devices, and their use in space technology for effective shielding and energy storage. It is part of the future, but the environmental impact needs to be studied before its widespread use. Furthermore, considering climate change, the depletion of fossil minerals, and sustainable farming, it is essential to adopt new methods. This manuscript investigates drought and boron (B) nanoparticles’ (NPs) effects on pea plants. It is known that B is necessary for plants of the leguminous family because it makes nitrogen fixation more efficient. In addition, B plays a crucial role in the formation and stability of cell walls, supports the functional and structural integrity of biological membranes, promotes the movement of sugar or energy to the growing parts of plants, and positively affects pollination and seed sets.

B is also known to stimulate both enzymatic and non-enzymatic antioxidant activity. Many scientific publications highlight the benefits of bulk B on the plant antioxidant system during different stress conditions [1,2,3], but only a few investigate the effect of B_2_O_3_ NPs on plants [4,5,6].

Recent research has shown that the application of 150 mg L^−1^ arbuscular mycorrhiza (AM) with 100 mg L^−1^ B_2_O_3_ NPs can significantly increase the height, the number of leaves, the fresh and dry biomass, and the herb g plant^−1^ of stevia [5]. It also positively affected the content of chlorophyll, carotenoids, and the amount of nutrients N, P, K, Zn, and B in stevia leaves. Another critical study highlighted that B_2_O_3_ NPs in soybean increased the B content, grain yield, and nitrogen accumulation compared to untreated plants [4]. In addition, B_2_O_3_ NPs have a positive effect in protecting potatoes against salinity stress. It increased the shoot fresh and dry weight, chlorophyll content, photosynthesis rate, stomatal conductance, intercellular CO_2_ concentration, and water use efficiency and decreased the transpiration rate in saline-soil-grown tomatoes [6].

One crucial consequence of B deficiency is abnormal nodular organogenesis, in which abnormal cell proliferation is accompanied by a lack of differentiation [7]. An adequate amount of boron can positively affect the formation and activity of antioxidants in plants [8]. Moreover, boron can increase plants’ resistance to salinity [3], heavy metals [9,10], and drought stress [11,12,13]. It is worth noting that there is no information on the response of drought-stressed plants to B as a nanoparticle. It was hypothesized that B NPs would likely increase the antioxidant content and activity in drought-stressed peas and reduce biomarkers of oxidative stress, thereby maintaining pea yields.

Peas are incorporated in crop rotation and are essential plants because they carry out symbiosis with nitrogen-fixing bacteria and enrich the soil quality, and they are important as a source of protein in nutritional aspects. Pea plants are sensitive to water scarcity, which makes them less attractive to farmers. To make pea cultivation more attractive to growers, it is extremely important to create a cultivation methodology that would support a constant yield. Considering the strong effect of bulk B on plants, it is very important to investigate the effect of B_2_O_3_ NPs on the growth parameters, yield, nodulation, antioxidants, and oxidative stress of drought-stressed peas for stronger effects and the more efficient use of depleted resources.

## 2. Materials and Methods

### 2.1. Research Conditions

The experiments were carried out during the spring–summer periods of two years (2019–2020) in two greenhouses (3 × 6 m; h = 2 m) at the Lithuanian Research Centre for Agriculture 41 and Forestry, Institute of Horticulture, Babtai, Lithuania (55°05′08.4″ N 23°48′03.5″ E, at an altitude of 51 m; moderate climate zone of the northern hemisphere). Before sowing, green pea seeds were sterilized in 5% sodium hypochlorite (NaClO) solution for 15 min to ensure surface sterility [14] and rinsed gently with deionized water several times. Then, the seeds were soaked in water for 24 h. Ten seeds were sown in 10-L volume plastic pots (7 pots per treatment, arranged randomized) and filled with ~8 kg of soil mixture (volume of 7:1 soil to perlite ratio, respectively). The soil was heavy loam with a particle size distribution, pH 7.4 ± 0.1; concentration of humus—3.6 ± 0.1%; P_2_O_5_—243 ± 8 mg kg^−1^; K_2_O—348 ± 37 mg kg^−1^; NH_4_—4 ± 0.6 mg kg^−1^; NO_3_—22 ± 0.9 mg kg^−1^; SiO_2_—39 ± 0.8 mg kg^−1^; B—0.02 ± 0.001 mg kg^−1^ (after the experiment, the composition of the soil was also analyzed). Pea seedlings were thinned to 7 plants per pot five days after sowing. After 16 days of cultivation, the peas were fertilized with 7 g pot^−1^ ammonium nitrate (NH_4_NO_3_, Merck KGaA, Darmstadt, Germany). The peas were sprayed with fungicides because the green pea cultivar ‘Respect’ is more susceptible to powdery mildew, even when grown in a greenhouse. Pots were irrigated with water by a graduated cylinder daily to 80% of substrate moisture (SM) using a substrate moisture sensor (Delta-T devices, HH2 moisture meter, Cambridge, United Kingdom) for 35 days. Plants were grown under a natural-day-length photoperiod. The average day/night temperature was 22.2/14.4 °C; relative air humidity—58/77 ± 5% before exposure; during the ten days of drought treatment, the average day/night temperature was 25.4/16.6 °C, and the relative air humidity was 53/75 ± 5%, and data were measured throughout the experiment (Termio+ data logger, Lubawka, Poland) in the first year. The conditions of the second experiment were as follows: the day/night average temperature was 24.2/14.4 °C; relative air humidity—54/75 ± 5% before exposure; during the ten days of drought exposure, the day/night average temperature was 26.2/17.0 °C, relative air humidity was 50/73 ± 5%. After the peas reached the 40 BBCH growth stage [15], they were foliar sprayed until full wetting (ca. 14 ± 0.5 mL plant^−1^) or watered (100 ± 1 mL per pot) with suspensions containing 12.5 ppm, 25 ppm, and 50 ppm concentrations of B_2_O_3_ NPs; control (NP-untreated) were watered or sprayed with water. After the application of NPs, the watering of one part of the pea plants was stopped, and drought stress was initiated (30% SM). Substrate moisture was measured every day at the same time and maintained at 30% by watering when needed. In contrast, another part of the pea and control plants was irrigated with water to maintain regular soil moisture (80% SM) throughout the experiment. These regimes were applied for ten days until harvest. After each treatment, plants were harvested after reaching the BBCH 50 growth stage [15] to assess their morphophysiological responses. The remaining plants were grown to maturity and harvested, the pods collected, and the grains counted and weighed.

### 2.2. Aqueous Suspension of Boron Nanoparticles

An aqueous suspension was prepared using boron (B_2_O_3_ particle size: up to 100 nm; purity: 95%) nanoparticles (US Research Nanomaterials, Inc, Houston, TX, USA) and deionized water at 12.5, 25, and 50 ppm concentrations. Before treatment with nanoparticles, the suspension was dispersed using an ultrasonic bath (Sonerex super ultrasonic bath 80W, Weidinger GmbH, Gernlinden, Germany) for 60 min. The stability of this suspension was evaluated using a particle size meter (Delsa™ Nano Submicron Particle Size, Beckman Coulter Instruments. Corporation, Fullerton, California) and a zeta potential device (Dispersion Technology Inc., Bedford Hills, New York). The pH of the suspension was determined using a pH meter (Hanna instruments, HI5000, Washington, USA).

### 2.3. Research Object

Green pea (*Pisum sativum* L.) cultivar ‘Respect’ (Maribo Seed International ApS, Denmark) was used in experiments. It is a medium–early semi-leafless pea variety. Green peas (*Pisum sativum* L.) were selected as the research object. Peas are the most drought-sensitive members of the legume family and have particular importance in crop rotation. In addition, they fix atmospheric nitrogen in a symbiotic association with *Rhizobium* bacteria and meet the nitrogen demand of subsequent crops. They are also widely used for both human food and animal feed.

### 2.4. Growth Parameters

Ten plants per treatment were randomly selected (n = 10) for biometric measurements. First, the shoots were separated from the roots, and then the shoot height, root length, fresh weight (FW), and dry weight (DW) were determined. Using electronic scales (Mettler Toledo AG64, Columbus, OH, USA), the FW and DW were measured. DW was determined using a forced-air convection dryer (VENTICELL 222, MBT, Brno, Czech Republic) at 105 ºC. After shoot FW determination, ten matured plants per treatment were floated on deionized water for 24 h, and turgid weights (TW) were measured. Relative water content (RWC) was calculated as described by [16].

(1) $$\mathrm{RWC},\;\%=\frac{\left(\mathrm{FW}\;-\;\mathrm{DW}\right)}{\left(\mathrm{TW}\;-\;\mathrm{DW}\right)}\times 100\;$$

The leaf area was measured with an automatic leaf area meter (AT Delta-T Devices, Wallingford, UK) and expressed as cm^2^ g ^−1^. Specific leaf area (SLA) was calculated by dividing the total plant leaf area (n = 10) by shoot DW. The root/shoot ratio was determined as the ratio of root DW to aboveground DW. Pods were collected from each pea plant, and the average number of heads/pods m^−2^ was counted (A, average number of pods m^−2^). Then, the number of grains in the pods in each variant was calculated, and the average (B, average number of grains per pod) was derived. The weight of 100 grains (C, weight of 100 grains of peas) was calculated. The pea yield was calculated according to the following formula [17]:

The pea yield (t ha^−1^) = (A × B × C)/10,000 (2)

### 2.5. Biochemical Analysis

Antioxidant properties of pea leaves were evaluated as the DPPH (2-diphenyl-1-picrylhydrazyl), ABTS (2,20-azino-bis (3-ethylbenzothiazoline-6-sulphonic acid)) diammonium salt, and radical scavenging activities, and the Fe^2+^ reducing antioxidant power assay (FRAP). Moreover, the total content of phenolic compounds was determined. Extracts were prepared by grinding 0.3 g of plant leaves with liquid nitrogen and diluting this with 5 mL of 80% methanol. Then, 24 h later, the samples were centrifuged for 10 min at 3000 rpm (Hermle Z300K, Baden-Württemberg, Germany). Cellulose filters were used for extract filtration. The supernatant was used for further analyses. All biochemical analysis was performed in 3 biological replications. Each of the three biological replicates consisted of at least three conjugated plants and was repeated in three analytical replicates.

#### 2.5.1. Non-Enzymatic Antioxidant Activity

The total content of phenolic compounds was determined as gallic acid equivalents. First, a 250 µL aliquot of the sample extract was mixed with 250 µL of 10% (*w*/*v*) Folin–Ciocalteu reagent, 500 µL of 1 M Na_2_CO_3_ solution, and 2 mL of distilled water [18]. After 20 min incubation in the dark, the absorbance was measured at 765 nm (M501, Spectronic Camspec Ltd., Leeds, UK). The total phenolic compound quantity mg g^−1^ was calculated from the calibration curve of gallic acid (0.01–0.1 mg mL^−1^, R^2^ = 0.99).

The ABTS (2,2-azino-bis (3-ethylbenzothiazoline-6-sulphonic acid; Sigma-Aldrich, Burlington, MA, USA) radical cation was obtained by incubating 7 mM ABTS stock solution (100 mL) with 2.45 mM potassium persulfate (final concentration K_2_S_2_O_8_; 99% purity; Sigma-Aldrich, Burlington, MA, USA) and allowing the mixture to stand in the dark at room temperature for 12–16 h before use [19]. After this, 50 μL of the prepared sample was mixed with 2 mL of ABTS solution (ABTS stock solution was diluted 1:7), and the absorbance was measured after 11 min (plateau phase) at 734 nm (M501, Spectronic Camspec Ltd., Leeds, UK). The ABTS scavenging activity of pea leaf extracts was calculated as the difference between the initial absorbance and after reacting for 10 min. A calibration curve was determined using Trolox (6-hydroxy-2,5,7,8-tetramethychroman-2-carboxylic acid; 97% purity; Sigma-Aldrich, USA) as an external standard, with a range of concentrations from 0.1 to 0.8 mM (R^2^ = 0.99). It was expressed as ABTS µmol scavenged per 1 g of fresh weight (µmol g^−1^ FW).

For the DPPH (2-diphenyl-1-picrylhydrazyl) assay, a stable 126.8 μM DPPH (100% purity; Sigma-Aldrich, Burlington, MA, USA) solution was prepared in methanol [20]. Subsequently, 1 mL of the DPPH solution was transferred to a test tube and mixed with 100 μL of the diluted pea extract with 400 μL methanol. The absorbance was scanned at 515 nm (M501, Spectronic Camspec Ltd., Leeds, UK). while reacting for 16 min. The free radical scavenging capacity was expressed as μmol of DPPH radicals scavenged per 1 g of fresh weight (µmol g^−1^ FW). A calibration curve was determined using Trolox (6-hydroxy-2,5,7,8-tetramethychroman-2-carboxylic acid; 97% purity; Sigma-Aldrich, USA) as an external standard, with a range of concentrations from 0.1 to 0.6 mM (R^2^ = 0.99).

The FRAP method is based on reducing ferric ions (Fe^3+^) to ferrous ions (Fe^2+^). The fresh working solution was prepared by mixing 300 mM, pH 3.6 acetate buffer, 10 mM TPTZ (2,4,6-tripyridyl-s-triazine) solution in 40 mM HCl, and 20 mM FeCl_3_ × 6H_2_O at 10:1:1 (*v/v/v*) [21]. Next, 20 µL of the sample was mixed with 3 mL of working solution and incubated in the dark for 30 min. Readings of the colored product (ferrous tripyridyl-triazine complex) were then taken at 593 nm. A calibration curve was determined using Fe_2_(SO_4_)_3_ (iron (III) sulfate; 97% purity; Sigma-Aldrich, USA) as an external standard, with a range of concentrations from 0.005 to 0.5 mM (R^2^ = 0.99). The antioxidant power is expressed as Fe^2+^ antioxidant capacity (Fe^2+^ µmol g^−1^ FW).

#### 2.5.2. Enzymatic Antioxidant Activity

The extracts used to determine the enzymatic antioxidant activity in pea leaves were prepared by grinding 0.5 g of fresh sample with liquid nitrogen and diluting within 5 mL extraction buffer (100 mM potassium phosphate buffer, pH 7.8, containing 0.1 mM EDTA). After centrifugation for 10 min at 3000 rpm (Hermle Z300K, Baden-Württemberg, Germany), the supernatant was collected and used for the assays of enzymatic activity. All steps in the preparation of the enzyme extract were carried out at 4 °C.

The dye-binding method and bovine serum albumin as a standard were used to determine soluble proteins. First, 30 µL of enzyme extract was mixed with 1.5 mL of Bradford reagent diluted by 1:5 with DI water. Absorbance was read after 2 min through a spectrophotometer (M501, Spectronic Camspec Ltd., Leeds, UK) at 595 nm [22].

Superoxide dismutase (SOD) activity was estimated by the inhibition of the photochemical reduction of nitroblue tetrazolium (NBT) by the enzyme [23]. Here, 3 mL of reaction mixture consisted of 13 mM methionine, 75 µM NBT, 100 mM potassium phosphate buffer (pH 7.8, containing 0.1 mM EDTA), 50 µL enzyme extract, and 13 μM riboflavin. The tubes were kept under 150 µmol m^−2^ s ^−1^ for 1 min to initiate the reaction and then covered. The absorbance was recorded after 30 min with a spectrophotometer (M501, Spectronic Camspec Ltd., Leeds, UK) at 560 nm, and one unit of enzyme activity was taken as the amount of enzyme that reduced the absorbance reading to 50% in comparison with tubes lacking the enzyme, expressed as unit mg^−1^ protein min^−1^.

Catalase (CAT) activity was measured as the disappearance of H_2_O_2_ [24]. First, 100 µL enzyme extract was added to 1.275 mL of 0.1 M phosphate buffer (pH 7.8, containing 0.1 mM EDTA). The reaction was started by adding 125 µL of 30 mM H_2_O_2_ (30%, Merck KGaA, Darmstadt, Germany). The decrease in absorbance measured by a spectrophotometer (M501, Spectronic Camspec Ltd., Leeds, UK) at 240 nm was observed for 1 min, and enzyme activity was computed by calculating the amount of H_2_O_2_ decomposed (µmol H_2_O_2_ mg^−1^ protein min^−1^).

Ascorbate peroxidase (APX) activity was assayed by recording the decrease in optical density due to ascorbic acid at 290 nm [25]. The 1 mL assay mixture contained 0.1 M potassium phosphate buffer (pH 7.8, containing 0.1 mM EDTA), 0.5 mM ascorbic acid, 0.1 mL enzyme extract, and 0.1 mL of 30 mM H_2_O_2_ (30%, Merck KGaA, Darmstadt, Germany) which was added to initiate the reaction. The decrease in absorbance was measured spectrophotometrically (M501, Spectronic Camspec Ltd., Leeds, UK) for 1 min. The extinction coefficient of 2.8 mM^−1^ cm^−1^ for reduced ascorbate was used to calculate the enzyme activity, which was expressed as µmol AsA mg^−1^ protein min^−1^.

Glutathione reductase (GR) activity was measured based on the decrease in the absorbance of oxidized glutathione (GSSG) at 340 nm [26]. The reaction mixture contained 0.1 M potassium phosphate buffer (pH 7.8, containing 0.1 mM EDTA), 1 mM GSSG (Merck KGaA, Darmstadt, Germany), 100 µL enzyme extract, and 75 µL 0.1 mM NADPH added last to initiate the reaction. The decrease in absorbance measured by a spectrophotometer (M501, Spectronic Camspec Ltd., Leeds, UK) was recorded every 5 min for 20 min. An absorption coefficient of 6.22 mM^−1^ cm^−1^ was used for calculations. GR activity was defined as µmol NADPH mg^−1^ protein min^−1^.

Guaiacol peroxidase (GPX) activity measurements were based on the increase in the absorbance of oxidized guaiacol at 470 nm [27]. The reaction mixture contained 0.1 M potassium phosphate buffer (pH 7.8, containing 0.1 mM EDTA), 31 mM guaiacol (Merck KGaA, Darmstadt, Germany), 100 µL enzyme extract, and 75 µL 3.6 mM H_2_O_2_ (30%, Merck KGaA, Darmstadt, Germany) added last to initiate the reaction. The increase in absorbance measured by a spectrophotometer (M501, Spectronic Camspec Ltd., Leeds, UK) was recorded for 2 min. Therefore, GPX activity was expressed as µmol H_2_O_2_ mg ^−1^ protein min^−1^.

#### 2.5.3. Oxidative Stress Biomarkers

The extracts used to determine the concentration of lipid peroxidation and hydrogen peroxide (H_2_O_2_) in pea leaves were prepared by grinding 0.1 g of fresh sample with liquid nitrogen and diluting it with 4 mL of 0.1% trichloroacetic acid (TCA). After centrifugation for 10 min at 3000 rpm (Hermle Z300K, Baden-Württemberg, Germany), the supernatant was used for further analyses.

For H_2_O_2_ measurements in plant leaves, 500 μL of the supernatant was added to 1 mL of 1 M potassium iodide (KI). The absorbance of the mixture was scanned at 390 nm using a spectrophotometer (M501, Spectronic Camspec Ltd., Leeds, UK). A calibration curve was determined using H_2_O_2_ (30%, Merck KGaA, Darmstadt, Germany) as an external standard, with a range of concentrations from 0.6 to 24.3 mM (R^2^ = 0.99). The content of H_2_O_2_ is expressed as fresh weight (µmol g ^−1^ FW) [28].

The thiobarbituric acid (TBARS) test determines the malondialdehyde (MDA) content in pea leaf samples as the end product of lipid peroxidation. First, 500 μL of the supernatant was added to 1 mL 0.5% (*w*/*v*) thiobarbituric acid (TBA, Merck KGaA, Darmstadt, Germany) in 20% trichloroacetic acid (TCA, Merck KGaA, Darmstadt, Germany). The mixture was incubated in boiling water for 30 min. The reaction stopped after the samples had cooled. The samples were centrifuged (Hermle Z300K, Baden-Württemberg, Germany) at 10,000× *g* for 5 min, and the absorbance of the supernatant was measured at 532 nm using a spectrophotometer (M501, Spectronic Camspec Ltd., Leeds, UK). The value for non-specific absorbance at 600 nm was subtracted [29]. The amount of MDA–TBA complex (red pigment) in leaves was calculated and expressed as nmol g ^−1^ FW:

C_MDA_ = (A_532_ − A_600_)/E_MDA_ (3)

C_MDA_—concentration of MDA, µM;
A_532_, A_600_—absorbance at wavelength;
E_MDA_—MDA extinction coefficient 155 mM^−1^ cm^−1^.

### 2.6. Elemental Composition Analysis

The macro- and microelement quantities in pea leaves, stems, and roots were determined using the microwave digestion technique combined with inductively coupled plasma optical emission spectrometry [30,31]. Complete digestion of dry plant material (0.3 g) was achieved with 8 mL 65% HNO_3_ using a microwave digestion system, Multiwave GO (Anton Paar GmbH, Graz, Austria). The digestion program was as follows: (1) 170 °C reached within 3 min, digested for 10 min; (2) 180 °C reached within 10 min, digested for 10 min. Fully digested samples were diluted to 50 mL with deionized water. The elemental profile was analyzed using an ICP–OES spectrometer (Spectro Genesis, SPECTRO Analytical Instruments, Kleve, Germany). The operating conditions employed for ICP–OES determination were 1300 W RF power, 12 L min^−1^ plasma flow, 1 L min^−1^ auxiliary flow, 0.8 L min^−1^ nebulizer flow, and 1 mL min^−1^ sample uptake rate. The analytical wavelengths chosen were P I 213.618 nm, K I 766.491 nm, S I 182.034 nm, Ca II 445.478 nm, Mg II 279.079 nm, Fe II 259.941 nm, Zn I 213.856 nm, Mn II 259.373 nm, Cu I 324.754 nm. The calibration standards were prepared by diluting a stock multi-elemental standard solution (1000 mg L ^−1^) in 6.5% (v/v) nitric acid and by diluting stock phosphorus and standard sulfur solutions (1000 mg L^−1^) in deionized water. The calibration curves for all the studied elements were in the range of 0.01–400 mg L^−1^. The levels of macro- and microelements in the dry weight of pea are presented.

### 2.7. Statistical Analysis

All the values were presented as mean ± standard deviation. Data were analyzed using the Analysis of Variance (ANOVA) test followed by Tukey’s HSD at *p* ≤ 0.05 to identify significant differences (XLStat software, Addinsoft, Paris, Ile-de-France, France, 2022). Differences were analyzed between variants when peas were grown under normal conditions and separately between peas grown under drought conditions. The micro- and macroelement analysis results were also compared between controls, i.e., peas grown in a drought and not treated with NPs (SM 30%) and treated with NPs, and between control peas grown under normal conditions (SM 80%) not treated with NPs and treated with NPs.

## 3. Results

### 3.1. Boron Nanoparticles’ Impact on Morphological Parameters

Pea height increased by 14 and 27% when watered and by 28 and 19% when sprayed with 12.5 and 50 ppm B_2_O_3_ NP suspension under sufficient substrate moisture (Table 1, 80% SM). Furthermore, a positive effect of B_2_O_3_ NPs was found in the pea plant’s leaf area and RWC. At the same time, a decrease of 15% in SLA was observed after spraying with 12.5 ppm solution. The root-to-shoot ratio statistically reliably increased after watering plants at 12.5 ppm by 21%, 25 ppm—by 36%, and 50 ppm—by 68%. In addition, an increase in the root-to-shoot ratio by 68% (12.5 ppm), 18% (25 ppm), and 34% (50 ppm) was observed when plants were sprayed. The results also showed that B_2_O_3_ NPs positively affected the number of nodules on plant roots by increasing their amount by up to 5.6 times when plants were watered and up to 3.4 times when plants were sprayed. The results showed that pea irrigation with 50 ppm B_2_O_3_ NPs had a significant positive effect on yield, while foliar treatment increased the pea yield for suspensions containing 12.5 and 25 ppm B_2_O_3_ NPs.

B_2_O_3_ NPs strongly affected pea plants grown in drought conditions (Table 1, 30% SM). The applied B_2_O_3_ NP suspension with different concentrations increased the plant height. Furthermore, watering the plants with 25 and 50 ppm B_2_O_3_ NP suspensions increased the leaf area by 30 and 40%, respectively. There was a statistically significant increase in RWC at higher B_2_O_3_ NP concentrations. The root-to-shoot ratio increased to 30% after watering drought-affected peas with B_2_O_3_ NP solutions, while foliar application increased the ratio to 14%. The 50 ppm B_2_O_3_ NP concentration influenced the number of root nodules, increasing it by three times during watering and up to six times during spraying. Irrigation with the suspension of 12.5 and 25 ppm B_2_O_3_ NPs positively affected the pea yield. Additionally, spraying drought-stressed peas with 12.5, 25, and 50 ppm B_2_O_3_ NPs increased the yield by 16%.

### 3.2. Effects on Oxidative Stress Biomarkers

**Figure 1:**
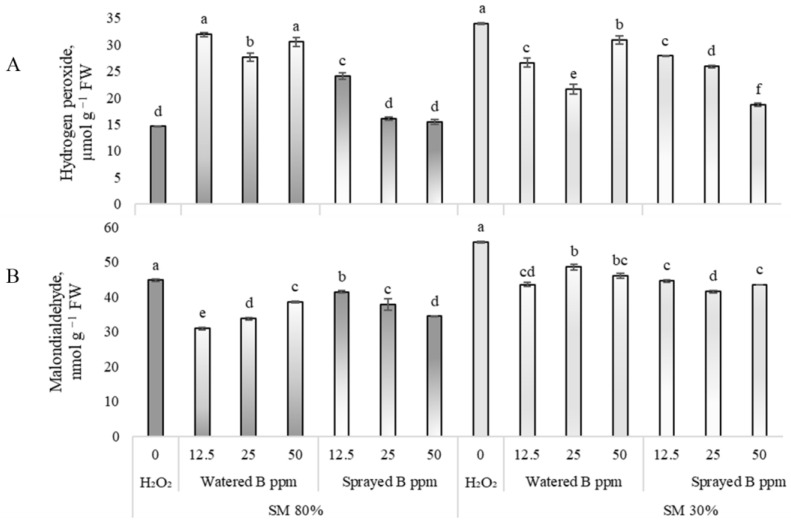
Effects of drought and B_2_O_3_ NPs (B in the figure; 12.5, 25, 50 ppm) on hydrogen peroxide (**A**) and malondialdehyde content (**B**) in *P. sativum* L. Substrate moisture (SM) 80% means normal conditions; drought stress—SM 30%. H_2_O—control plants watered with deionized water. Values presented are means ± SE of three replicates, and different letters differed significantly by Tukey’s HSD test (*p* < 0.05).

The results show that exposure to B_2_O_3_ NPs through the roots increased the amount of H_2_O_2_ in plants, regardless of the concentration, when peas were grown under sufficient substrate moisture (Figure 1A, 80% SM). When plants were sprayed, a statistically reliable 65% increase in H_2_O_2_ content was found at 12.5 ppm B_2_O_3_ NPs. A significant decrease in the MDA concentration (Figure 1B, 80% SM) was also found in pea leaves as plants were watered or sprayed with a solution containing any concentration of B_2_O_3_ NPs.

The significant inhibition of H_2_O_2_ and MDA was found as their concentration decreased after plants’ exposure to drought and B_2_O_3_ NPs (Figure 1A, B 30% SM). The amount of H_2_O_2_ decreased by 18, 24, and 45% after spraying the plants with 12.5, 25, and 50 ppm, and by 22, 37, and 9% after watering. A reduction in the MDA content by 22, 13, and 17% was found after pea irrigation with 12.5, 25, and 50 ppm suspensions of B_2_O_3_ NPs and after foliar application by 20, 25, and 22%.

### 3.3. Effects on Non-Enzymatic Antioxidants

**Figure 2:**
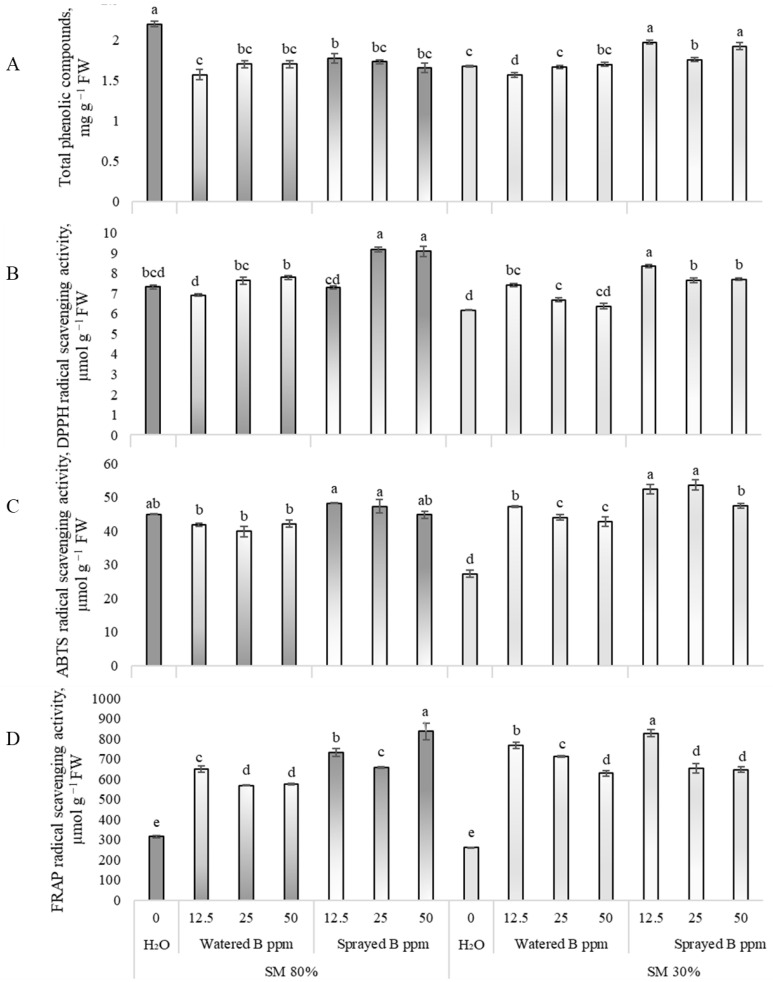
Effects of drought and B_2_O_3_ NPs (B in the figure; 12.5, 25, 50 ppm) on total phenolic compounds (**A**), DPPH free radical scavenging activity (**B**), ABTS free radical scavenging activity (**C**), and FRAP antioxidant power (**D**) in *P. sativum* L. Substrate moisture (SM) 80% means normal conditions; drought stress—SM 30%. H_2_O—control plants watered with deionized water. Values presented are means ± SE of three replicates, and different letters differed significantly by Tukey’s HSD test (*p* < 0.05).

It was found that at 80% substrate moisture, watering and spraying with B_2_O_3_ NPs reduced the TPC in pea leaves by up to 30% (Figure 2A, 80% SM). B_2_O_3_ NP treatment did not affect ABTS free radical scavenging activity (Figure 2C, 80% SM). However, it was determined that after spraying peas with 25 and 50 ppm suspensions, the DPPH free radical scavenging activity increased by 25 and 24% (Figure 2B, 80% SM). Furthermore, concentrations of B_2_O_3_ NP suspensions of 12.5, 25, and 50 ppm increased the FRAP antioxidant power (Figure 2D, 80% SM), as plants were watered or sprayed.

The results showed that spraying drought-affected peas with 12.5, 25, and 50 ppm B_2_O_3_ NP suspensions increased the TPC content to 18%, while watering with 12.5 ppm significantly reduced it (Figure 2A, 30% SM). ABTS free radical scavenging activity showed sensitivity to the impact of B_2_O_3_ NPs (Figure 2C, 30% SM); it increased to 73% after watering and 96% after spraying compared to drought-affected plants without NP exposure. Similar results were found for FRAP antioxidant power in peas (Figure 2D, 30% SM). The exposure to drought and B_2_O_3_ NP 12.5 and 25 ppm suspensions through the roots exerted a slight impact (20%) on DPPH free radical scavenging activity (Figure 2B 30% SM). In addition, spraying with 12.5, 25, and 50 ppm B_2_O_3_ NP suspensions induced DPPH free radical scavenging activity by 35, 24, and 25%, respectively.

### 3.4. Effects on Enzymatic Antioxidants

**Figure 3:**
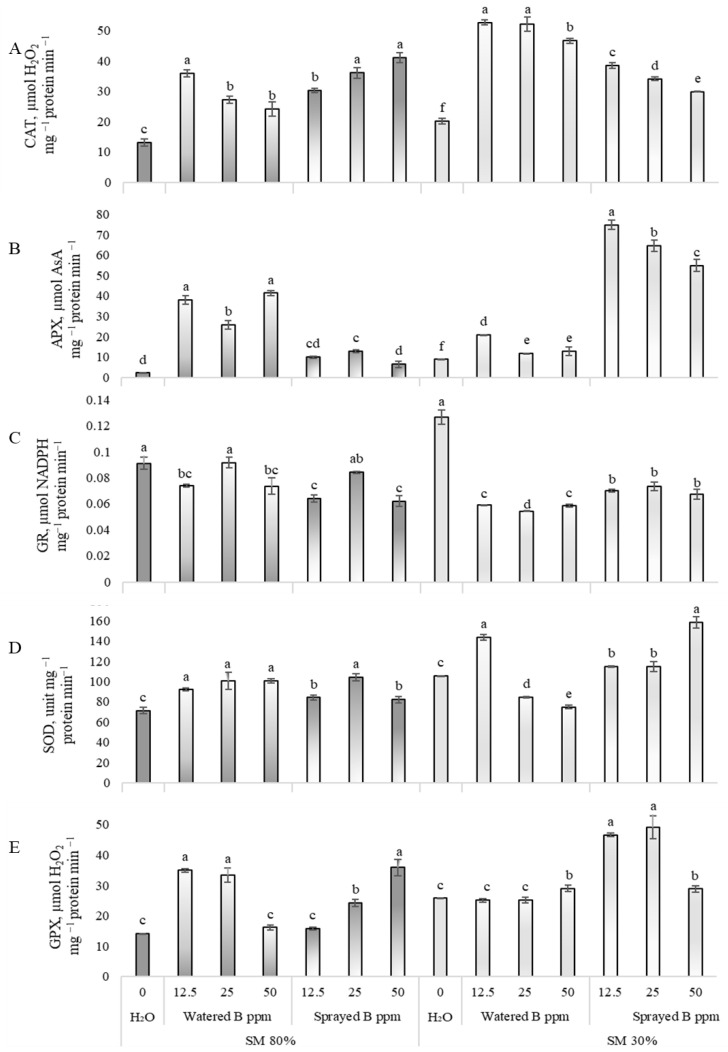
Effects of drought and B_2_O_3_ NPs (B in the figure; 12.5, 25, 50 ppm) on ascorbate peroxidase (APX, **A**), catalase (CAT, **B**), superoxide dismutase (SOD, **C**), glutathione reductase (GR, **D**), and guaiacol peroxidase (GPX, **E**) activity in *P. sativum* L. Substrate moisture (SM) 80% means normal conditions; drought stress—SM 30%. H_2_O—control plants watered with deionized water. Values presented are means ± SE of three replicates, and different letters differed significantly by Tukey’s HSD test (*p* < 0.05).

B_2_O_3_ NPs induced the activity of CAT, APX, SOD, and GPX in pea leaves when they were grown in 80% SM (Figure 3A,B,D,E). APX activity increased particularly strongly after watering plants with B_2_O_3_ NP suspensions, while a slightly weaker effect was caused by spraying. CAT activity increased up to two times when plants were watered with suspensions of B_2_O_3_ NPs. When peas were sprayed, the CAT activity increased by 1.3, 1.8, and 2 times when the concentration was 12.5, 25, and 50 ppm. SOD activity was induced by up to 41% by exposure to B_2_O_3_ NPs through roots, and foliar treatment activated the enzyme by up to 46%. GPX activity was distinguished because lower concentrations of 12.5 and 25 ppm had a more substantial positive effect during watering, while higher concentrations of 25 and 50 ppm increased the activity more strongly during spraying. The B_2_O_3_ NP suspension reduced the GR activity (Figure 3C, 80% SM) when suspensions with concentrations of 12.5 and 50 ppm were used for plant watering or spraying.

A substantial decrease in GR activity was caused by drought and B_2_O_3_ NP exposure, with a 55% reduction after irrigation and a 45% reduction after spraying (Figure 3C, 30% SM). Moreover, an adverse effect was found on SOD activity (Figure 3D, 30% SM); after peas’ irrigation with 25 and 50 ppm solutions of B_2_O_3_ NPs, a 36% increase in SOD activity was determined after using the 12.5 ppm B_2_O_3_ NP suspension. Furthermore, SOD activity was induced by up to 51% when drought-affected peas were sprayed with the B_2_O_3_ NP solution. The strong effect of B_2_O_3_ NPs on the APX activity (Figure 3B, 30% SM) remained in pea leaves as plants were grown in drought conditions. After watering peas with B_2_O_3_ NP suspensions, APX activation occurred by up to 1.4 times after spraying up to eight times. Additionally, GPX activity in drought-affected peas (Figure 3E, 30% SM) was increased by up to 91% after foliar exposure with all B_2_O_3_ NP concentrations. CAT activity (Figure 3A, 30% SM) was strongly activated by the watering or spraying of plants with B_2_O_3_ NP solutions of any concentration.

### 3.5. Comparison and Summary of Results

As can be seen in the heat map (Table 2), in peas grown with normal substrate moisture and watered or sprayed with B_2_O_3_ NPs, nodulation, FRAP, hydrogen peroxide formation, GPX, APX, and CAT were most strongly induced, but ABTS antioxidant capacity, TPC and MDA content, and GR activity were reduced. Strong nodulation; increased ABTS antioxidant capacity, FRAP antioxidant power, GPX, APX, and CAT activity; and significantly decreased H_2_O_2_ and MDA levels were observed in peas grown under drought conditions with B application. Notably, nodule formation increased with an increasing concentration, but the activity and content of most antioxidants decreased, although they were still higher than in plants grown under drought conditions but without B_2_O_3_ NPs. The yield of peas grown under drought conditions was higher with the corresponding increase in antioxidant content and activity, which means that as the concentration of B_2_O_3_ NPs decreased, the yield increased, and it even increased by up to 19% compared to B_2_O_3_-untreated plants.

## 4. Discussion

In our studies, the zeta potential of the aqueous suspension of B_2_O_3_ NPs was −28.54 mV (Table 1). However, other researchers found that an aqueous suspension of B_2_O_3_ NPs with 0.2% Triton X-100 had a value −30.3 mV [32]. Such zeta potential values indicate that the solutions are stable and anionic. In addition, the PDI of this suspension was 0.23, while other scientists have found a value of 0.4 [32], indicating that the suspensions are monodisperse.

To better understand the effects of B on plants, it is important to determine the form and mechanism with which B enters the plant naturally. Around 96% of boron exists mainly as boric acid (H_3_BO_3_) and a small amount as borate anion [B(OH)_4_^−^] at a neutral pH of 5.5–7.5 [33]. Such forms of B can readily diffuse through roots or be transported by major intrinsic protein (MIP) channels or BOR transporters depending on the plant species [34].

The study shows that the number of nodules formed on pea roots (Table 2) increases strongly after exposure to B_2_O_3_ NPs in both regular and deficient substrate moisture conditions. Such an effect could be explained by the fact that once B enters the plant, it is transported in the xylem, and approximately 90% is incorporated into plant cell walls [35]. B is the main element in the formation of esters with rhamnogalacturonan II (RGII). This borate ester is required to maintain normal cell wall functions and structures [36]. Under normal conditions, when there is sufficient B in the soil, RGII glycoproteins are also formed in the plasma membranes of pea root nodules and root cells. However, RGII glycoproteins are not synthesized, and their absence destabilizes the plasma membrane and nodule formation in B deficiency [37]. Moreover, B, as a component of glycoproteins, is essential for differentiating nodule bacteria into a nitrogen-fixing form [38]. Increased nitrogen fixation in the pea plant increases its resistance, resulting in increased antioxidant production, as seen in our results (Table 2).

It is worth mentioning that our study expands the knowledge about the effect of B_2_O_3_ NPs on the antioxidant systems of plants. It should be emphasized that B_2_O_3_ NPs effectively protect plants from drought stress by stimulating non-enzymatic FRAP antioxidant power and ABTS free radical scavenging activity and APX, GPX, and CAT enzymatic antioxidants. Moreover, B NPs can also influence the shoot height, flower number, and B, N, and K accumulation in soybeans [4]. Furthermore, algae treated with B NPs showed higher Chl content and MDA and H_2_O_2_ concentrations, and more active SOD and CAT enzymes [39]. In addition, B deficiency affected >70% of the analyzed genes [7]. Most were upregulated, but some genes critical for nodule development and function were downregulated.

NPs can help to maintain the pea yield even under adverse environmental conditions. Their effect depends on the concentration and method of exposure. B_2_O_3_ NPs have a positive effect on the antioxidant system of peas, reducing the amount of oxidative stress biomarkers, which positively affects growth indicators, but more detailed studies are needed to evaluate their overall effects on different ecosystems. These findings contribute to the application of nanoparticles in agronomy but we do not recommend their use in practice until their effects on different ecosystems are studied.

## 5. Conclusions

Spraying with B_2_O_3_ nanoparticles at 12.5 ppm most effectively stimulated phenol accumulation, antioxidant capacity, ascorbate peroxidase, superoxide dismutase, and guaiacol peroxidase enzyme activity in pea leaves exposed to drought. In addition, B_2_O_3_ nanoparticles reduced the amount of hydrogen peroxide and malondialdehyde in pea plants grown on a substrate with insufficient moisture. The most substantial positive effect was found on peas affected by drought after spraying them with 12.5 ppm of B_2_O_3_ nanoparticles. These findings contribute to the application of nanoparticles in agronomy, but we do not recommend their use in practice until their effects on different ecosystems are studied.

## Figures and Tables

**Table 1:** Impact of drought stress and 12.5, 25, 50 ppm B_2_O_3_ NPs on *P. sativum* L. leaf area, height, relative water content (RWC), root-to-shoot ratio, specific leaf area (SLA), number of nodules, and yield. 0—control plants watered with deionized water; substrate moisture (SM) 80%; drought stress—SM 30%. Mean values within columns followed by different letters differ significantly at *p* < 0.05 (n = 10) according to Tukey’s (HSD) test.

B_2_O_3_ NPs, ppm			Leaf Area, cm^2^	Plant Height, cm	RWC, %	Root/Shoot Ratio	SLA, m^2^ kg ^−1^	Number of Nodules	Yield, t ha^−1^
SM 80%		0	36.1 c	28.4 d	82.5 d	7.8 c	5.3 ab	1.7 d	3.9 b
	Watered	12.5	46.1 a	32.4 bc	84.3 c	9.4 b	6.1 a	11.0 a	3.1 c
		25	39.2 bc	30.3 cd	86.1 ab	10.5 b	5.1 ab	5.0 c	4.0 ab
		50	46.8 a	36.1 a	86.8 ab	13.1 a	4.7 ab	9.7 ab	4.7 a
	Sprayed	12.5	49.9 a	36.3 a	87.3 a	13.1 a	4.5 b	7.3 bc	4.4 a
		25	44.9 ab	30.9 bcd	85.5 bc	9.2 b	5.0 ab	5.7 c	4.3 a
		50	44.6 ab	33.8 ab	85.6 bc	10.4 b	5.1 ab	5.0 c	3.9 b
SM 30%		0	33.1 b	26.0 e	53.0 c	9.2 c	5.0 a	2.0 c	2.5 c
	Watered	12.5	27.5 b	28.4 d	52.6 c	9.6 b	4.2 ab	2.3 c	2.9 a
		25	42.9 a	32.7 a	58.3 ab	10.5 a	4.8 a	6.0 b	2.7 ab
		50	46.4 a	30.4 bc	59.1 a	11.9 a	4.2 ab	8.3 b	2.6 bc
	Sprayed	12.5	31.6 b	29.3 cd	51.9 c	10.2 a	4.4 ab	2.0 c	3.0 a
		25	33.4 b	31.2 ab	55.2 bc	10.5 a	3.9 ab	3.3 c	2.9 a
		50	31.2 b	29.6 cd	57.8 ab	9.6 b	3.5 b	13.7 a	2.9 a

**Table 2:** The impact of drought stress and B_2_O_3_ NPs (12.5, 25, 50 ppm) on *P. sativum* L. grown in a substrate with sufficient (SM 80%) and insufficient (SM 30%) moisture is expressed as a percentage change (%) compared to the control (for SM, 80% control means plants grown under SM 80% and NP-untreated; SM 30% control means drought-affected but NP-untreated plants) in the heat map. Statistically significant differences are marked in bold.

Treatment B_2_O_3_ NPs, ppm	SM 80%						SM 30%					
	Watered			Sprayed			Watered			Sprayed		
	12.5	25	50	12.5	25	50	12.5	25	50	12.5	25	50
Plant height	**14**	7	**27**	**28**	9	**19**	**9**	**26**	**17**	**13**	**20**	**14**
Leaf area	**28**	9	**30**	**38**	**25**	**24**	−17	**30**	**40**	−4	1	−6
Nodules	**560**	**200**	**480**	**340**	**240**	**200**	17	**200**	**317**	0	67	**583**
RWC	**2**	**4**	**5**	**6**	**4**	**4**	−1	**10**	**11**	−2	4	**9**
Root/shoot	**21**	**36**	**68**	**69**	**18**	**34**	**4**	**14**	**30**	**11**	**14**	**5**
SLA	14	−5	−11	−15	−6	−3	−17	−4	−16	−13	−23	**−29**
Yield	**−21**	2	**20**	**12**	**11**	−1	**16**	**10**	6	**19**	**16**	**14**
ABTS	−7	−11	−6	7	5	0	**73**	**61**	**56**	**92**	**96**	**74**
DPPH	−5	4	7	0	**25**	**24**	**20**	**8**	3	**35**	**24**	**25**
TPC	**−29**	**−22**	**−22**	**−19**	**−21**	**−25**	**−6**	−1	1	**18**	**5**	**15**
FRAP	**106**	**81**	**83**	**132**	**109**	**166**	**194**	**174**	**141**	**217**	**151**	**148**
HP	**119**	**89**	**109**	**65**	10	6	**−22**	**−37**	**−9**	**−18**	**−24**	**−45**
MDA	**−31**	**−25**	**−14**	**−7**	**−15**	**−23**	**−22**	**−13**	**−17**	**−20**	**−25**	**−22**
GR	**−19**	0	**−19**	**−30**	−8	**−32**	**−53**	**−57**	**−54**	**−45**	**−42**	**−47**
GPX	**147**	**136**	14	11	**71**	**153**	−3	−2	**13**	**81**	**91**	**12**
APX	**1657**	**1100**	**1817**	363	**498**	200	**136**	**33**	**46**	**750**	**634**	**522**
SOD	**29**	**41**	**41**	**18**	**46**	**15**	**36**	**−20**	**−29**	**9**	**9**	**51**
CAT	**173**	**107**	**84**	**131**	**175**	**214**	**161**	**159**	**132**	**91**	**69**	**48**

RWC—relative water content, SLA—specific leaf area, TPC—total phenolic compounds, HP—hydrogen peroxide, MDA—malondialdehyde, GR—glutathione reductase, GPX—guaiacol peroxidase, APX—ascorbate peroxidase, SOD—superoxide dismutase, CAT—catalase. 0—control plants watered with deionized water, drought stress—30% substrate moisture.

## Data Availability

Not applicable.
